# Exploring Chemical Markers Related to the Acceptance and Sensory Profiles of Concentrated Liquid Coffees: An Untargeted Metabolomics Approach

**DOI:** 10.3390/foods11030473

**Published:** 2022-02-05

**Authors:** Mónica Quintero, Maria José Santander, Sebastián Velásquez, Julián Zapata, Mónica P. Cala

**Affiliations:** 1 Research and Development Center—Colcafé S.A.S., Medellín 050024, Colombia; svelasquez@colcafe.com.co; 2 Metabolomics Core Facility—MetCore, Vice-Presidency for Research, Universidad de los Andes, Bogotá 110111, Colombia; mj.santander10@uniandes.edu.co (M.J.S.); mp.cala10@uniandes.edu.co (M.P.C.); 3 Instituto de Química, Universidad de Antioquia, Medellín 050010, Colombia; julian.zapatao@udea.edu.co

**Keywords:** untargeted metabolomics, concentrated liquid coffee, sensory analysis, high-performance liquid chromatography, mass spectrometry

## Abstract

In this study, we aimed to apply an untargeted LC/QTOF-MS analysis for the identification of compounds that positively and negatively affect the acceptance of coffee beverages from liquid coffee concentrates (CLCs) before and after storage. The metabolomic results were integrated with physicochemical and sensory parameters, such as color, pH, titratable acidity, and oxygen contents, by a bootstrapped version of partial least squares discriminant analysis (PLS-DA) to select and classify the most relevant variables regarding the rejection or acceptance of CLC beverages. The OPLS-DA models for metabolite selection discriminated between the percent sensory acceptance (the Accepted group) and rejection (the Rejected group). Eighty-two molecular features were considered statistically significant. Our data suggest that coffee sample rejection is associated with chlorogenic acid hydrolysis to produce ferulic and quinic acids, consequently generating methoxybenzaldehydes that impact the perceived acidity and aroma. Furthermore, acceptance was correlated with higher global scores and sweetness, as with lactones such as feruloyl-quinolactone, caffeoyl quinolactone, and 4-caffeoyl-1,5-quinolactone, and significant oxygen levels in the headspace.

## 1. Introduction

Coffee is the third most consumed beverage globally, preceded only by water and tea. In 2020, despite the economic impact of the pandemic, 166 million sacks of coffee were consumed worldwide [1]. In countries such as the USA and Japan, most coffee categories depicted growth: 21 and 32% for the instant coffee category with market values close to 1000 and 4900 MUSD in the USA and Japan, respectively; 23 and 19% for the roasted and ground coffee category with market values of 14,700 and 5477 MUSD, respectively; and in the iced/ready-to-drink coffee segment (RTD), 11% growth and a 14% decline with market values of 10,600 MUSD and 13,900 MUSD, respectively. Despite the decline in the RTD category in Japan, its value remains remarkable compared to that of most traditional categories, such as roasted and ground coffee or instant coffee [2]. The RTD category has seen more than 140 launches during the past year, with more than 10% of these claiming either distinctive sensory profiles associated with the extraction process (cold brew) or another flavor attribute related to premiumization of the product. These new products require exceptional flavor and aroma, a brand-new experience, and an extended shelf life that is compatible with new consumption habits [2].

The coffee market has a deep appreciation for Colombian coffee due to its soft palatability, delicate acidity, and intense aroma. Colombian coffee has more acidity, citrus-like and fruity red notes, and a soft taste. Sometimes, the postharvest process imparts dedicated fermented notes to the cup, which is a unique characteristic of washed coffees. The emergence of the specialty coffee segment has motivated science to examine the chemical phenomena occurring among coffee cultivars and their origin and postharvest and posterior processing to produce highly appealing products with special sensory profiles [3]. The understanding of the sensory profiles of coffee beverages has migrated from hedonic attributes that assess the product’s quality to descriptors that provide detailed information on products that meet the quality criteria required by processing but are specific for certain markets. However, the identification of chemical markers related to the sensory quality of coffee products has been a challenge [4,5,6]. The roasted and ground coffee segment has widely incorporated this sensory description of the products, but this is not the case for instant coffee or concentrated extracts, as coffee bean and posterior extract processing imparts features that diverge from the traditional profile expectation.

Moreover, the chemical composition of coffee beverages comprises a wide range of families of substances present in different concentrations. To date, more than 1000 compounds have been reported [7,8]. Numerous studies have been conducted to understand the impact of chemical composition on the final coffee beverage, including origin [9,10,11], cultivar [12,13], postharvest processing [11], and industrialization [6,14]. Prior to 2006, most of these studies utilized multivariate statistical methods to explain the variability of the complex data, accompanied by instrumental analytical methods with conventional detectors, known as targeted analysis. Nevertheless, this approach does not provide sufficient resolution power to explain the overall variation and complete correlation with the sensory attributes to assess the shelf life of the new products.

The perception of coffee quality is mainly driven by its aroma, while acceptance of the beverage is mostly related to the perceived taste. The hedonic attributes usually used to assess the product do not discriminate across the chemical fingerprints of the samples, while the analytical platforms available for study do not provide sufficient granularity for specific compound and reaction identification. Novel techniques including metabolomics allow a better understanding of the chemical coffee composition in different matrices, such as green coffee, coffee-based beverages, and roasted and ground coffee. The application of untargeted metabolomics approaches in food science has emerged as a powerful tool to explore the complete set of metabolites in these matrices, known as food metabolomics or foodomics [14,15,16,17,18].

In recent years, food metabolomics has been applied to all stages of food systems, from farms to industrial food processing and food intake [19]. Untargeted metabolomics analyses of coffee have been carried out to identify the coffee’s origin [11], correlation with sensory quality before roasting [12], roasting process [4,6,20,21], beverage and extraction methods [11,20], and instant coffees [6]. However, there have not been enough metabolomics studies to find the chemical markers that are associated with sensory rejection of coffee drinks from liquid coffee concentrates (CLCs) that have spent time in storage. In our previous paper, we performed a targeted analysis to describe the dependence of CLC acceptance based on decrement of the sensory quality and how these attributes correlate with changes in the chlorogenic acid and carbohydrate contents [21].

Nevertheless, the chemical mechanisms involved in sensory deterioration are unknown because of the complex composition of CLCs as coffee products. Evaluation of this complexity has been used as an approach to understand the chemical reactions related to sensorial spoilage of CLCs and to develop mitigation strategies. Similarly, the chemical markers responsible for quality loss and taste differences in CLCs are currently not fully understood. If the chemical markers and reaction mechanisms are identified, these studies might be used to design mitigation alternatives for shelf life extension.

As reported in previous studies, the most critical attribute associated with CLC spoilage is the aroma and increased perceived acidity. Aroma reductions occur due to reactions between chlorogenic acids (CGAs), particularly 5-CQA and 3-CQA, and available oxygen to form quinic acid, ferulic acid, hydroxyhydroquinone (HHQ), and other related compounds [22,23,24,25]. Previous studies have demonstrated a possible correlation between aroma reduction and the interaction of oxygen with mercaptofurans in coffee beverages [25,26]. The development of undesirable acidity during coffee beverage storage has been suggested due to the hydrolysis of chlorogenic acid lactones and changes in the release of chlorogenic acids linked to melanoidins [27,28,29]. Other studies have argued that these reactions generate changes in the aroma profile, imparting spicy and phenolic notes from methoxy phenols as products of these chemical mechanisms of degradation [29,30]. Metabolomics has been implemented to investigate some coffee applications (green and roasted coffee) but has not yet been considered for CLCs.

This study’s objective was to compare the sensory acceptance of beverages produced from CLCs on an industrial scale and then stored for at least six months at room temperature with their frozen controls to determine correlations between their physicochemical properties. Thus, an untargeted metabolomics approach was applied to select the main metabolites and identify possible chemical markers associated with CLC sensory quality and acceptance. The metabolomic results were integrated into physicochemical and sensory parameters such as color, pH, titratable acidity, and oxygen contents by a bootstrapped version of partial least squares discriminant analysis (PLS-DA) to select and classify the most relevant variables regarding the rejection or acceptance of CLC beverages. The results showed that the identified metabolites allow the assessment of possible deteriorative reactions involving chlorogenic acids and their related compounds and methoxybenzaldehydes. Changes in these kinds of substances were correlated with variations in the aroma and acidity attributes. Overall, these findings agree with some reports by other authors on coffee beverages.

## 2. Materials and Methods

### 2.1. Coffee Samples

CLC samples were prepared from coffee roasted to be mild-dark (CIELab L* = 25.00 ± 0.4) in a food processing plant that produces instant coffee (Colcafé S.A.S., Medellín, Colombia). The process involved a percolation battery with six extractors, each loaded with 300 kg of coffee and fed 180 °C steam under constant flow for extraction. Then, the liquid was processed by freeze-concentration to reach a concentration of 35–37% TDS. In this part of the process, the concentration increased in the range of 10–15% TDS. Finally, to avoid microbiological spoilage, the CLC was pasteurized at ultrahigh temperature (121 °C for 5 s). The package consisted of an aseptic bag with the following characteristics: 2 L capacity and a double-layer film with an external film layer composed of polyethylene (PE)/polyethylene terephthalate (PET)/PE and an internal layer composed of PE/ethylene-vinyl-alcohol (EVOH). The samples were stored under two different temperature conditions, at 25 °C or frozen at −30 °C, for 120 days for analysis to achieve sensory differences due to deterioration changes.

The specifications for the transmission rate in this package system were as follows: oxygen transmission of 0.16 cc/m^2^/day at 25 °C and water vapor permeability of 11 g/m^2^/day. Before storing the samples, the contents of oxygen in the headspace of the packages and the portion dissolved in the extract were determined.

### 2.2. Untargeted Metabolomic Analysis

#### 2.2.1. Metabolomic Analysis by RP-LC-QTOF-MS

First, 40 mg of each sample was taken for metabolite extraction; these values were adjusted according to the total dissolved solids (TDS). Then, each sample was mixed with 820 µL of Type I water, vortexed for 5 min, and then placed in an ultrasonic bath for 5 min. After that, the samples were centrifuged at 16,000 rpm and 4 °C for 10 min. A mixture of 20 µL of the supernatant and 180 µL of Type I water was transferred to an Eppendorf tube for further analysis. Analysis was performed using an Agilent Technologies liquid chromatography–quadrupole time-of-flight–mass spectrometry (LC–QTOF–MS) system (Agilent Technologies, Waldbronn, Germany). The extract was injected onto an InfinityLab Poroshell 120 EC-C18 column (3 × 100 mm 2.7 µm, Agilent, CA, USA) maintained at 30 °C. The flow rate of the mobile phase (A: Milli-Q water with 0.1% formic acid (*v*/*v*), B: acetonitrile with 0.1% formic acid (*v*/*v*)) was 0.4 mL/min. The gradient elution started at 2% B, increased to 98% B over 19 min, and ended by going back to the initial conditions in 1 min, where it was held for 5 min to allow column re-equilibration. During both analyses, two reference masses were used and continuously infused into the system for constant mass correction: *m*/*z* 121.0509 (C_5_H_4_N_4_) and *m*/*z* 922.0098 (C_18_H_18_O_6_N_3_P_3_F_24_) for positive ionization mode (ESI+) and *m*/*z* 112.9856 [C_2_O_2_F_3_ (NH_4_)] and *m*/*z* 1033.9881 (C_18_H_18_O_6_N_3_P_3_F_24_) for negative ionization mode (ESI−). The mass spectrometry system was operated in full-scan mode from 50 to 1100 *m*/*z*. Data were collected in centroid mode at a scan rate of 1.00 spectrum per second, the capillary voltage was set to 3000, the drying gas flow rate was 8 L/min at 325 °C, the gas nebulizer was set to 50 psi, the fragmentor voltage was 175 V, the skimmer was 65 V, and the octupole radio frequency voltage (OCT RF Vpp) was set to 750 V for both positive and negative ionization modes.

#### 2.2.2. Quality Control (QC) Samples

The reproducibility of sample preparation and the stability of the LC–MS system were evaluated by employing QC samples. QC samples were prepared by pooling equal volumes of each extracted sample, and then 10 QC samples were injected at the beginning of the analysis to equilibrate the chromatographic system, after every five randomized coffee samples, and at the end of each sample sequence.

#### 2.2.3. Data Treatment

Deconvolution, alignment, and integration were performed using algorithms such as Molecular feature extraction and Recursive feature extraction in Agilent MassHunter Profinder B.10.0 software. After that, a manual inspection was performed, aiming to clean up background noise and unrelated ions. Finally, the acquired data were exported to Excel for filtering by presence and reproducibility, keeping only the metabolites present in 100% of the samples in at least one group while maintaining a coefficient of variation in the QC samples of less than 20%.

#### 2.2.4. Statistical Analysis

To evaluate statistically significant differences between the metabolomic profiles of the groups, univariate statistical analysis (UVA) and multivariate statistical analysis (MVA) were performed using SIMCA 16.0 (Umetrics, Umea, Sweden) and MATLAB (R2019b, Mathworks, Inc., Natick, MA, USA), respectively. First, MVA based on principal component analysis (PCA) was applied to evaluate the acquired data quality, verifying that the QC samples were correctly clustered in these models to guarantee the stability of the analytical system. After that, orthogonal partial least square–discriminant analysis (OPLS-DA) models were built to maximize and inspect the differences between the study groups and select responsible metabolites for group separation. Pareto scaling was used for transformation before statistical analysis. For UVA, data normality was verified by evaluating the Kolmogorov–Smirnov, Lilliefors, and Shapiro–Wilk tests and the variance ratio by Levene’s test. The *p* value was determined parametrically (unpaired *t* test) or nonparametric (Mann–Whitney U test) with Benjamini and Bonferroni–Hochberg false discovery rate post hoc correction (FDR). For data from both ionization modes, the significant variables were selected by keeping only those that fulfilled the following parameters: (1) UVA (*p* value with Benjamini–Hochberg FDR of <0.05); (2) MVA criteria (variable importance in projection (VIP) of >2 with jack-knife confidence interval (JK) not including the zero value from OPLS-DA with CV-ANOVA of <0.05); and (3) change percent of >50%.

#### 2.2.5. Metabolite Identification

Accurate masses of statistically significant features were searched using the CEU Mass Mediator tool (http://ceumass.eps.uspceu.es/, accessed on 15 June 2021) with databases such as METLIN (http://metlin.scripps.edu, accessed on 15 June 2021), KEGG (www.genome.jp, accessed on 15 June 2021), HMDB (http://hmdb.ca, accessed on 15 June 2021), and Lipid MAPS (http://lipidmaps.org, accessed on 15 June 2021). Finally, some metabolite identities were confirmed by LC−MS/MS.

### 2.3. Integration of the Physicochemical and Sensory Properties

#### 2.3.1. pH and Titratable Acidity

The pH and titratable acidity analyses were performed at 25 °C using a Mettler Toledo DL 22 pH meter automatic system (Columbus, OH, USA). Titratable acidity was determined by combining 100 mL of each beverage with 0.1 N NaOH until neutrality was reached (pH 7.00).

#### 2.3.2. Total Dissolved Solids (TDS) Content

The TDS content was determined by examining the relationship between the measured Brix value and a correction factor, representing the concentration of sucrose in the sample. The measurements were carried out using a Mettler Toledo R50 refractometer (Columbus, OH, USA).

#### 2.3.3. Color

Beverage color was measured using a Hunterlab D25 LT colorimeter (Reston, VA, USA). Before each measurement, the instrument was calibrated using white and green tiles. Color results are expressed as rectangular coordinates L*, a*, and b* in CIELab parameters, in which L* indicates the degree of luminosity of whiteness or blackness (from 0 to 100). In the chromatic portion of the color, a* represents color changes in the red (+a*) to green (−a*) ratio, and b* indicates the blue (−b*) to yellow (+b*) ratio. Hue describes the overall intensity, while chroma (saturation) may be defined as the strength or dominance of the hue. Equations (1) and (2) depict the calculation of both parameters.

(1) $$Hue=\pm arctan\left(\frac{b^{*}}{a^{*}}\right)$$

(2) $$Chroma=+\sqrt{{\left(a^{*}\right)}^{2}+{\left(b^{*}\right)}^{2}}$$

#### 2.3.4. Oxygen and Carbon Dioxide Contents

Measurements of the oxygen (O_2_) and carbon dioxide (CO_2_) contents in the headspace were made using a Moccon 325 analyzer (Minneapolis, MN, USA). The lower detection limit with this instrument was approximately 0.1%, and the resolution was 0.01%.

#### 2.3.5. Sensory Analysis

Sensory analysis was performed by a specialized panel from Colcafé S.A.S., with an age range of 30 to 55 years. Ten judges were trained in discriminative and descriptive testing for at least 100 h prior to the real analysis. Each analysis was performed in duplicate. The analyses were performed in individual cubicles at a maintained relative humidity and temperature of 50–65% and 25 °C, respectively, following the ISO 6658:2005 standard. Prior to the sensory sections, each panelist had at least 100 h of training. Data were acquired using Fizz sensory software V2.47.

Coffee beverages were prepared from a dilution of each CLC in hot water at 90 ± 2 °C at pH 7.0 to obtain a beverage with 2% TDS. The freshly prepared coffee brews were evaluated immediately. During the tasting sessions, 20 mL of coffee was served in a 50 mL odorless plastic cup at 70 °C. The samples were coded with randomized 3-digit numbers. Water was used for palate cleansing between samples.

The sensory evaluation comprised a descriptive test measuring the following attributes: aroma, acidity, bitterness, body, sweetness, winey flavor, and overall perception of the coffee beverages prepared from the CLCs. The intensity of each descriptor was scored on a scale from 0 to 10. Furthermore, in the second step, each panelist classified the samples into one of two groups: “Accepted”, representing that they had accepted the consumption of this product, and “Rejected”, representing rejection.

#### 2.3.6. Parameter Identification and Classification Model

Twelve samples (six accepted and six rejected) with three replicates each were analyzed. All considered parameters (i.e., the physicochemical and sensory properties and the oxygen, carbon dioxide, and chlorogenic contents of the samples) were considered for possible correlations and variable impacts on the classification of the accepted beverages. Before assembling the model, each of the parameters was individually analyzed to evaluate its capacity to discriminate the two groups (accepted and rejected beverages). For this univariate analysis, normality was evaluated by the Shapiro test for each feature, analysis of variance (ANOVA) was computed to feed the least mean squares mean estimation, and Tukey’s pairwise comparisons were carried out to compare both types of beverages for each attribute. When normality was not met, nonparametric Dunn tests were calculated for median comparison and group discrimination.

After the univariate analyses, a bootstrapped version of PLS-DA was assessed for classification and variable impact evaluation. Consequently, 100 training sets were randomly sorted and stratified by the CLC code, in which 2 of the biological samples were used for training and the whole dataset was used for model validation. This approach was considered not to evaluate model accuracy (i.e., sensitivity and specificity evaluation) but instead to identify variable impacts on each of the groups and possible correlations across the feature types. For variable selection, both the VIP and selectivity ratio (SR) were considered. After sorting the latter in descending order, those variables that did not meet a specific threshold (1 for VIP and 4 for SR) were removed from the model in forward stepwise optimization. Whenever the classification accuracy was maintained or decreased, the variable being tested was removed from the feature pool. This was performed for all variables that met the criteria. Cross validation with 5 samples was computed for latent variable number selection. For representation, a mean score plot with 96% confidence ellipses was constructed for the first two latent variables. The loading plot was considered a bubble dispersion plot, in which the bubble size depicts the selectivity ratio and, hence, the variable’s capacity to represent the variance of the dataset evaluated.

## 3. Results and Discussion

### 3.1. Untargeted Metabolomics by LC-QTOF-MS

**Figure 1:**
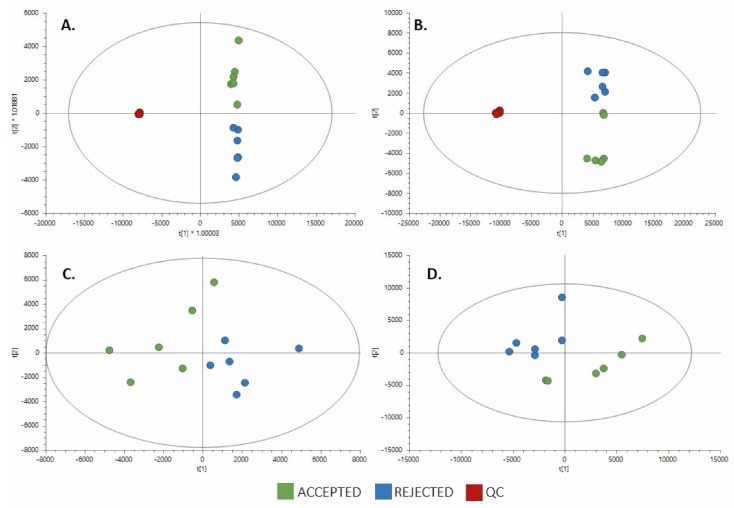
PCA score plots for untargeted metabolomics in both positive and negative ESI modes. (Green dots, accepted samples (*n* = 6); blue dots, rejected samples (*n* = 6); dark red dots, quality control samples, QC.) (**A**) LC-QTOF-MS (+) with QC samples: R^2^_(cum)_: 0.853, Q^2^_(cum)_: 0.847. (**B**) LC-QTOF-MS (−) with QC samples: R^2^_(cum)_: 0.873, Q^2^_(cum)_: 0.841. (**C**) LC-QTOF-MS (+): R^2^_(cum)_: 0.629, Q^2^_(cum)_: 0.386. (**D**) LC-QTOF-MS (−): R^2^_(cum)_: 0.701, Q^2^_(cum)_: 0.428.

In this study, we performed global metabolomics analysis using LC-QTOF-MS in both positive and negative ESI modes to obtain the broadest range of metabolites from concentrated liquid coffee. After data processing and filtering, the total numbers of features found were 814 from positive ESI mode and 687 from negative ESI mode. To evaluate the quality of the analytical platform, a PCA was built for each analysis. The clear clustering of QC samples in the unsupervised PCA models (Figure 1A,B) evidenced the stability and quality of the acquired data for both ESI analysis modes; therefore, this result supports that the separation between the groups is related to real biological differentiation. PCA also showed a clear separation between the samples that did and did not achieve acceptance (Accepted and Rejected groups) (Figure 1C,D).

**Figure 2:**
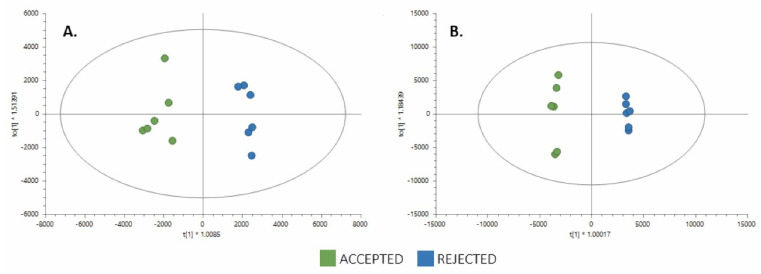
Comparison of the supervised OPLS-DA models for untargeted metabolomics of rejected samples (*n* = 6) vs. accepted samples (*n* = 6). (Green dots, accepted samples (*n* = 6); blue dots, rejected samples (*n* = 6)). (**A**): Positive ESI mode comparison LC-QTOF-MS (+): R2X(cum): 0.394, R2Y(cum):0.0965, Q2 (cum): 0.691, CV-ANOVA: 0.056. (**B**) Negative ESI mode—LC-QTOF-MS (−): R2X(cum): 0.868, R2Y(cum): 0.997, Q2 (cum): 0.974, CV-ANOVA: 0.00063.

After assuring data quality, UVA and supervised MVA were performed to obtain the differences between the groups. The differentiation of samples in the Accepted and Rejected groups was achieved using an OPLS-DA model (Figure 2). The OPLS-DA score plots showed evident separation of the groups. Acceptable values of the explained variance (R^2^), the predicted variance (Q^2^), and the CV-ANOVA were achieved from both analyses. Univariate analysis (UVA) was performed to assess the significance of each metabolite separately for comparison. The parameters to select the metabolites that were statistically significant in both ESI modes were those that met the following criteria: *p* value of <0.05 or VIP of >1, with JK intervals not containing zero from OPLS-DA with CV-ANOVA of <0.05 and change percent of >15%. The metabolites that met these requirements were identified as putative, confirmed, or unknown and are presented in Table 1.

As listed in Table 1, a total of 80 metabolites were determined to be statistically significant for this comparison. Most of the difference between the Accepted and Rejected groups corresponded to benzoic acids and their derivates, flavonoids, amino acid derivates, and other organic acids.

### 3.2. Integration of Untargeted Metabolomics with Sensory and Physicochemical Properties

For a comprehensive understanding of the interactions among the sensory attributes, the acceptability of the coffee beverages, and their chemical composition, univariate analysis was applied to each variable type, and the statistically significant selected features were parameters such as the color (a*, b*, hue, and chroma), titratable acidity, and oxygen and carbon dioxide contents; these data are presented in Table 2.

Table 2 supports the assertion that neither the pH nor the CLC concentration determine CLC acceptance. In contrast, titratable acidity and color variables such as a* (variation from green to red), chroma, and hue were also characteristic of each group. Rejected samples were characterized by a high titratable acidity and an a* value that had a tendency toward green. Furthermore, the luminosity of the concentrates did not have an effect on acceptance. Samples that had a high oxygen content in the headspace were associated with beverage acceptance, and those with increased carbon dioxide contents were mostly rejected.

**Figure 3:**
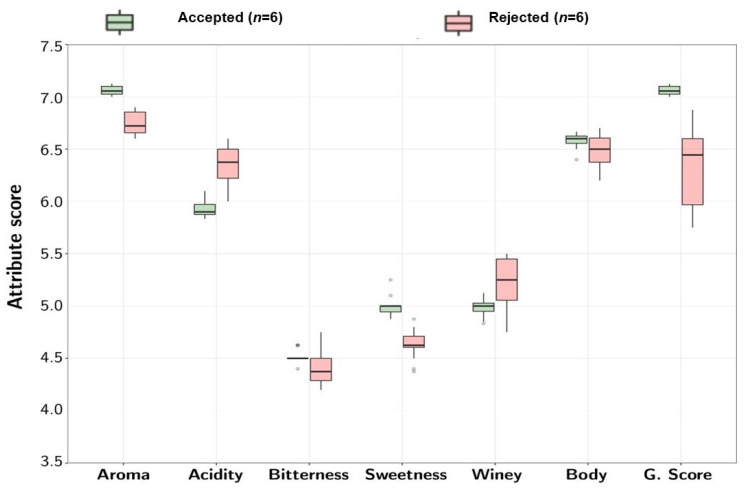
A box plot representing the sensory profiles of the evaluated samples to compare the acceptability associated with the Accepted and Rejected groups.

The dissolved oxygen content was not related to acceptance, while high oxygen saturation was associated with the rejected samples. Nevertheless, these measurements had considerable dispersion. The sensory attributes from each profile are described in Figure 3. A box plot depicts the dispersion across samples.

**Figure 4:**
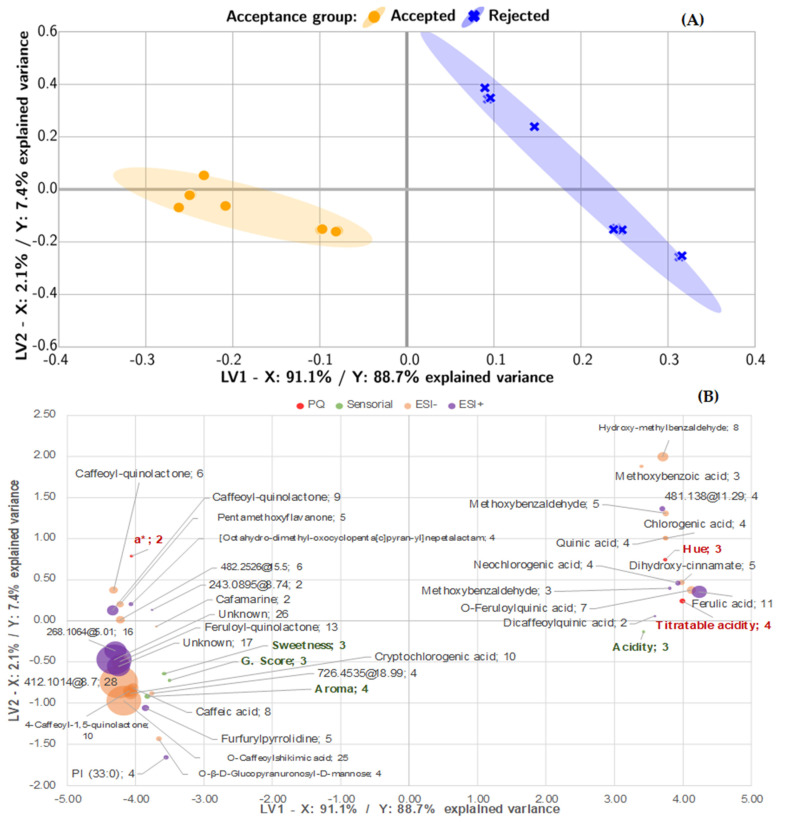
Bootstrapped PLS-DA biplot for acceptance classification. (**A**) Score plot for the averaged PLS-DA model for the first two latent variables, and (**B**) the loadings and selectivity ratio (bubble area) for the physicochemical, sensory, ESI−, and ESI+ datasets for the bootstrapped PLS-DA for acceptance classification.

Figure 3 portrays significant differences across the two groups for most of the attributes. Acceptance was associated with improved aroma, sweetness, and global score, while elevated acidity resulted in rejection. The bitterness, winey flavor, and body attributes did not show a specific trend in either of the groups. All statistically significant variables were included in the PLS-DA model. The scores model is presented in Figure 4A, and the loadings plot is presented in Figure 4B, which depicts the interactions across the evaluated parameters.

As with the multivariate metabolomics model, the classification achieved with the integrated PLS-DA model was adequate for beverage acceptance classification. In Figure 4A, Latent Variable 1 represented 91% of the variance for the X dataset, while it represented 89% of the variability for the Accepted group classification. In all cases, both groups were accurately classified. The second component represented 2% of the variance for X and 7% for the Accepted group. The left or negative side of LV1 was associated with acceptance, whereas the right side was related to rejection. The second component, LV2, contains samples that were strongly rejected on the negative side. The dispersion of rejection is distributed across the two latent variables, while the dispersion of acceptance is distributed across LV1.

Acceptance-related variables are on the negative side of the LV1 coordinate, and rejected variables are on the positive side. The molecular features with higher molecular weights (values over 336 a.m.u.), with the exception of caffeic acids, are lactones, esters with shikimic acid, and unknown compounds, which are concentrated on the left side of the plot; hence, they were correlated with acceptance. In contrast, molecular features on the positive axis LV1 were associated with strongly rejected samples and, in turn, were correlated with high and titratable acidity, ferulic, O-feruloylquinic and dicaffeoylquinic acids, and methoxybenzaldehyde. Intermediate rejection samples were characterized by elevated hue and high contents of chlorogenic and quinic acids.

When these results were contrasted with those in Table 1, ferulic acid showed greater variation (161) and was predominant in the Rejected group. Therefore, these findings agree with those reported in the literature that correlated variations in chlorogenic acids with beverage spoilage [20,24,25]. These chlorogenic acids are esters formed between quinic acid and trans-cinnamic acids (caffeic, p-coumaric, and ferulic acids) [24,31,32]. Specifically, the hydrolysis of feruloyl quinic acids in the presence of oxygen releases quinic acid and ferulic acid. In one pathway, the decarboxylation of ferulic acid (176.0473@10.57) acts as a precursor of the formation of methoxybenzaldehydes (MN_136.0523@11.33, MN_136.0527@6.29, MN_136.0524@8.42) [4,33]. The presence of these compounds might have a negative impact on the acidity and aroma attributes, as they are correlated to product rejection. Methoxybenzaldehydes are present in coffee brews; however, these compounds promote undesirable and unbalanced flavors at high concentrations. For example, 4-methoxyphenyl, 4-ethyl-2-methoxyphenol (4-ethylguaiacol), and 4-ethenylguaiacol are associated with phenolic and medicinal notes that are prone to result in rejection of the beverage [31]. According to the organoleptic properties of chlorogenic acids (CGAs), caffeoylquinic acids result in lower acidity than free acids such as quinic or caffeic acids. Moreover, dicaffeoylquinic acid studies have shown a strong correlation between these attributes and metallic or bitter flavors in beverages [32]. Hence, the higher carbon dioxide contents in the Rejected group could be a product of the decarboxylation reactions of CGAs.

Quinic acid can react in another, parallel pathway, inducing hydroxyhydroquinone, which reacts with thiols and benzylic thiols (such as FFT). These substances are highly aromatic (i.e., high aroma activity values) and associated with roast and coffee notes in coffee beverages. These reactions have been thoroughly studied and have been demonstrated to negatively impact the aroma [23,24].

Consequently, rejection of CLCs might be strongly associated with the degradation of CGAs that results in the release of ferulic, quinic, and caffeic acids to start three degradation pathways that promote changes in some of the sensory attributes, such as aroma and acidity. These results agree with those presented in a previous work [21] and by other studies [28,30,34].

In contrast, acceptance was mainly related to an enhanced aroma, high global scores, and sweetness. Unknown metabolites 412.1014@10.14 and 439.1845@5.94 with high explicative powers (i.e., high selectivity ratios) were also related to this group. Furthermore, several lactones promoted beverage acceptance, such as feruloyl-quinolactone, caffeoyl quinolactone, and 4-caffeoyl-1,5-quinolactone. Although the sensory attributes did not represent the same variance as the metabolites, their loadings were similarly distributed. None of the oxygen-related variables were considered by the model, possibly due to high dispersion. Nevertheless, this does not suggest that such variables are not explicative of the deterioration phenomena; in contrast, they are highly correlated to most of the identified pathways.

As depicted on the negative side of LV1, the Accepted group mostly contains features such as lactones, chlorogenic-acid-derived compounds, and other unknown compounds, such as 726.4535@18.99 with a higher molecular weight. Subsequently, lactones from chlorogenic acids are a product of the roasting process [32,35]. Recent studies found that compounds such as 3-O-caffeoyl-4-O-3-methylbutanoyl quinic acid and 3-O-caffeoyl-4-O-3-methylbutanoyl-1,5-quinide were present in high concentrations in beverages that were positively evaluated. The judges reported enhancements in the aroma, aftertaste, and overall score attributes associated with these features. Consequently, this result might indicate that no hydrolysis reactions had occurred, guaranteeing beverage acceptance.

## 4. Conclusions

An analytical strategy using untargeted metabolomics by LC/MS-QTOF and bootstrapped PLS-DA was applied in this work to discriminate between the acceptance and rejection of coffee beverages made from CLCs. Untargeted metabolomics analysis based on LC/MS-QTOF successfully discriminated between the features that affected the acceptance and rejection of CLCs. The correlations between chlorogenic acid hydrolysis and its impact on sensory attributes and its association with the rejection of coffee beverages were identified. Similarly, the presence of methoxybenzaldehydes as the products of these reactions suggests the importance of this pathway. Nevertheless, it is still unclear how all of the chemical components are involved and react during storage. Further research is required to understand the complex mechanisms of the deteriorative reactions in CLCs that affect consumer acceptance and the effects of lactone-type compounds. New studies that include the synthesis and recombination of these compounds, along with characterization techniques such as nuclear magnetic resonance (NMR) spectroscopy, might insightfully identify the reaction mechanisms during the deterioration of sensory quality in CLCs. Moreover, this accord elucidates the metabolites formed during storage, their impacts on sensory evaluation, and the identification of mitigation strategies during the deteriorative process. The current study is the first to contrast the composition and critical sensory attributes with CLC shelf life by metabolomics analysis to understand sensory deterioration and the chemical pathways involved.

## Figures and Tables

**Table 1:** Metabolites significantly differentiated between the Accepted and Rejected groups using both ESI modes by untargeted metabolomics via quadrupole time-of-flight mass spectrometry (GM-LC/MS-QTOF).

Compound	Formula	Mass	RT (min)	Mass Error (ppm)	Adduct	CVforQC (%) ^a^	DET	CON	Rejected vs. Accepted Samples		
									Change (%) ^b^	VIP ^c^	*p* Value ^d^
Cytosine	C_4_H_5_N_3_O	111.0433	1.59	7	M+H	3.3	ESI+	Putative	−55	1.5155	0.0086
Methylfuran-carboxylic acid	C_6_H_6_O_3_	126.0317	4.45	5	M+H-H_2_O	4.3	ESI+	Putative	−173	2.3476	0.0338 *
Octene-diynoic acid	C_8_H_6_O_2_	134.0368	7.01	4	M+H-H_2_O	1.8	ESI+	Putative	29	1.5942	0.0649
Hydroxy-methylbenzaldehyde	C_8_H_8_O_2_	136.0524	11.33	5	M-H	2.8	ESI−	Putative	61	1.6083	0.0275 *
Methoxybenzaldehyde	C_8_H_8_O_2_	136.0524	6.29	2	M-H	2.9	ESI−	Putative	17	1.7607	0.0275 *
Vinylcatechol	C_8_H_8_O_2_	136.0524	9.52	4	M-H	3.7	ESI−	Putative	49	1.8414	0.0086
Hydroxyphenethylamine	C_8_H_11_NO	137.0841	11.52	4	M+H-H_2_O	6.3	ESI+	MSMS	82	1.0769	0.0151
Benzofurancarboxaldehyde	C_9_H_6_O_2_	146.0368	8.27	4	M+H	1.4	ESI+	Putative	29	1.4441	0.0411
Cinnamic acid	C_9_H_8_O_2_	148.0524	8.95	5	M+H	1.6	ESI+	Putative	44	1.2417	0.0151
(Furanyl)-tetrahydropyridine	C_9_H_11_NO	149.0841	1.57	5	M+H	3.4	ESI+	Putative	−97	1.5258	0.0510 *
Ethylnicotinate	C_8_H_9_NO_2_	151.0633	2.26	3	M+H	13.7	ESI+	Putative	−130	1.6802	0.0086
Furfurylpyrrolidine	C_9_H_13_NO	151.0997	6.29	4	M+H-H_2_O	5.8	ESI+	Putative	93	1.8865	0.0338 *
Methoxybenzoic acid	C_8_H_8_O_3_	152.0473	8.42	3	M+H-H_2_O	2.5	ESI+	Putative	109	1.6151	0.0510 *
Hydroxycoumarin	C_9_H_6_O_3_	162.0317	11.84	3	M-H	8.5	ESI−	MSMS	−92	3.4280	0.0275 *
Pyridoxal	C_8_H_9_NO_3_	167.0582	1.51	5	M+H	3.4	ESI+	Putative	90	1.6302	0.0338 *
Carboline	C_11_H_8_N_2_	168.0687	10.30	5	M+H	1.3	ESI+	Putative	−29	1.1236	0.0411
Isovalerylalanine	C_8_H_15_NO_3_	173.1052	2.60	16	M+Na	3.1	ESI+	Putative	−101	1.1844	0.0151
Methyl-quinolin-diol	C_10_H_9_NO_2_	175.0633	8.24	4	M+H	2.1	ESI+	Putative	−78	2.3966	0.0510 *
(Cyclohexylmethyl)pyrazine	C_11_H_16_N_2_	176.1313	3.53	1	M+K	1.8	ESI+	Putative	52	1.0421	0.0649
[2H-Pyrrol-(3H)-ylidenemethyl]-furanmethanol	C_10_H_11_NO_2_	177.0790	6.94	3	M+H-H_2_O	1.4	ESI+	Putative	−65	1.2231	0.0151
Dimethyl-(1-pyrrolidinyl)-cyclopenten-one	C_11_H_17_NO	179.1310	8.88	5	M+H	3.6	ESI+	Putative	−130	1.0109	0.0510 *
Caffeic acid	C_9_H_8_O_4_	180.0423	8.90	3	M+H-H_2_O	8.0	ESI+/−	Identified	37	1.0605	0.1320
Hydroxy-(hydroxyphenyl)propenoicacid	C_9_H_8_O_4_	180.0423	14.69	3	M+H-H_2_O	1.9	ESI+	Putative	−27	1.5147	0.1320
Indole-propionic acid	C_11_H_11_NO_2_	189.0790	11.14	4	M+H	4.9	ESI+	Putative	−102	1.3162	0.0338 *
Quinic acid	C_7_H_12_O_6_	192.0634	6.09	1	M-H	1.7	ESI−	MSMS	16	1.5511	0.0086
Ferulic acid	C_10_H_10_O_4_	194.0579	10.57	3	M+H-H_2_O	6.0	ESI+	Putative	161	1.5558	0.0338 *
(Furan+B63:B75yl)-hexahydro-7H-cyclopenta[b]pyridinone	C_12_H_13_NO_2_	203.0946	5.51	3	M+H	5.9	ESI+	Putative	−173	1.6442	0.0338 *
Methylsalicyluric acid	C_10_H_11_NO_4_	209.0688	1.33	3	M+H	5.2	ESI+	Putative	1918	2.5034	0.0338 *
1-Isothiocyanato-8-(methylthio)octane	C_10_H_19_NS_2_	217.0959	3.64	10	M+Na	5.5	ESI+	Putative	1011	1.6326	0.0338 *
1-Arabinofuranosylcytosine	C_9_H_13_N_3_O_5_	243.0895	4.40	20	M+H	9.1	ESI+	Putative	199	1.0998	0.0086
243,0895@8,74	-	243.0895	8.74	-	M+H	4.8	ESI+	Putative	−95	1.0254	0.0510 *
Hydroxy-(hydroxy-methyl-hexenyl)benzofuran	C_15_H_18_O_3_	246.1256	5.01	2	M+Na	2.8	ESI+	Putative	−328	2.5216	0.0338 *
N-Phenylacetylasparticacid	C_12_H_13_NO_5_	251.0794	3.12	2	M+H-H_2_O	5.2	ESI+	MSMS	−254	2.2707	0.0338 *
N-Pyruvoyl-methoxy-hydroxyanthranilate	C_11_H_11_NO_6_	253.0586	1.36	3	M+H	3.2	ESI+	Putative	1860	1.9896	0.0338 *
N,N’-Diphenyl-phenylenediamine	C_18_H_16_N_2_	260.1313	1.67	10	M+Cl	3.6	ESI−	Putative	25	1.0798	0.0086
268,1064@5,01 *	-	268.1064	5.01	-	M+H	5.6	ESI+	Putative	−328	2.5216	0.0338 *
Evoxanthidine	C_15_H_11_NO_4_	269.0688	4.40	7	M+H	3.5	ESI+	Putative	207	1.2801	0.0338 *
Trichostachine	C_16_H_17_NO_3_	271.1208	7.32	3	M+H	10.9	ESI+	Putative	218	1.4652	0.0338 *
Deaminofusarochromanone	C_15_H_19_NO_4_	277.1314	5.13	3	M+H	5.7	ESI+	MSMS	68	1.2220	0.0151
Eriodictyol	C_15_H_12_O_6_	288.0634	11.34	1	M-H	3.9	ESI−	MSMS	34	1.0096	0.0275 *
317,0903@3,49	-	295.1088	3.49	-	-	1.6	ESI+	MSMS	94	1.1685	0.0510 *
ent-Hydroxybuphanisine	C_17_H_19_NO_4_	301.1314	6.93	3	M+H	4.4	ESI+	Putative	205	1.9999	0.0338 *
291,1111@7,49	-	309.1212	7.49	-	M+H	4.2	ESI+	Putative	−189	1.1515	0.0411
O-p-Coumaroyl-D-glucose	C_15_H_18_O_8_	326.1002	5.74	1	M-H-H_2_O	2.6	ESI−	Putative	192	1.0547	0.0275 *
Guaiacin	C_20_H_24_O_4_	328.1675	17.17	2	M+H	6.5	ESI+	Putative	−197	2.3579	0.0338 *
331.215@13.47	C_20_H_29_NO_3_	331.2147	13.47	3	M+H	3.2	ESI+	Putative	−102	1.1645	0.0338 *
5-O-Caffeoylshikimicacid//4-Caffeoyl-1,5-quinolactone *	C_16_H_16_O_8_	336.0845	12.84	1	M-H	6	ESI−	Putative	−100	2.4478	0.0275 *
Caffeoyl-quinolactone	C_16_H_16_O_8_	336.0845	11.32	3	M+H	5.6	ESI+/−	Putative	−422	4.4402	0.0338 *
O-Caffeoylshikimicacid	C_16_H_16_O_8_	336.0845	11.84	0	M-H	2.1	ESI−	Putative	−87	5.6218	0.0275 *
Feruloyl-quinolactone *	C_17_H_18_O_8_	350.1002	13.86	1	M-H	5.8	ESI−	Putative	−82	3.3243	0.0275 *
Chlorogenic acid	C16H_18_O_9_	354.0951	8.42	1	M-H	2.7	ESI−	Identified	21	2.4680	0.0086
Cryptochlorogenic acid	C16H_18_O_9_	354.0951	9.75	2	M-H-H_2_O	2.1	ESI−	Identified	−77	2.9124	0.0275 *
Neochlorogenic acid	C16H_18_O_9_	354.0951	6.29	3	M+H	5.0	ESI+/−	Identified	106	1.1735	0.0086
O-beta-D-Glucopyranuronosyl-D-mannose	C_12_H_20_O_12_	356.0955	1.38	1	M-H-H_2_O	2.8	ESI−	Putative	−23	1.1169	0.0150
N-Caffeoyltryptophan	C_20_H_18_N_2_O_5_	366.4000	1.37	1	M-H	4.2	ESI−	Putative	−29	1.5959	0.0275 *
Piperundecalidine	C_23_H_29_NO_3_	367.2147	18.79	7	M+H	6.9	ESI+	Putative	−276	1.3874	0.0338 *
O-Feruloylquinicacid	C_17_H_20_O_9_	368.1107	8.64	0	M-H	3.7	ESI−	Putative	30	1.2937	0.0275 *
Pentamethoxyflavanone	C_20_H_22_O_7_	374.1366	18.72	1	M+FA-H	3.7	ESI−	Putative	−48	1.0967	0.0275 *
412.1014@8.70 *	C_31_H_12_N_2_	412.1000	8.70	1	M-H	3.6	ESI−	Putative	−73	1.1022	0.0275 *
439.1852@7.47//ValHisTyr	C_20_H_27_N_5_O_5_	417.2012	7.47	6	M+Na	14.3	ESI+	Putative	−543	1.8897	0.0338 *
ValHisTyr	C_20_H_27_N_5_O_5_	417.2012	5.94	6	M+Na	9.2	ESI+	Putative	−499	2.0702	0.0338 *
447,1498@4,01	-	447.1498	4.01	-	M+H	5.0	ESI+	Putative	−30	1.0433	0.4848
(+)-CatechinC-glucoside	C_21_H_24_O_11_	452.1319	16.70	2	M-H-H_2_O	3.9	ESI−	Putative	−39	1.0612	0.0275 *
481,138@11,29	-	459.1529	11.29	-	M+H	7.2	ESI+	Putative	14895	1.2221	0.0338 *
N-[(Dihydropterinyl)methyl]-(beta-D-ribofuranosyl)anilinephosphate	C_18_H_23_N_6_O_8_P	482.1315	3.91	8	M-H-H_2_O	2.0	ESI−	Putative	−31	1.2776	0.0275 *
482,2526@15,50	-	482.2526	15.50	-	M-H	2.7	ESI−	Putative	−26	1.4000	0.0275 *
498,3072@18,79	-	498.3072	18.79	-	-	2.1	ESI+	Putative	−245	2.7344	0.0338 *
Aconine	C_25_H_41_NO_9_	499.2781	12.24	3	M+H	2.5	ESI+	Putative	21	1.3575	0.2402
Galactomannan	C_18_H_32_O_16_	504.1690	1.59	1	M+H-H_2_O	2.9	ESI+	MSMS	−108	1.0085	0.1796
Cafamarine	C_26_H_36_O_10_	508.2308	17.19	0	M+FA-H	5.3	ESI−	Putative	−38	1.6678	0.0275 *
Dicaffeoylquinicacid	C_25_H_24_O_12_	516.1268	7.92	2	M+H	6.3	ESI+	MSMS	65	1.2450	0.0338 *
Di-O-caffeoylquinicacid//Dicaffeoylquinicacid	C_25_H_24_O_12_	516.1268	8.91	3	M+H	4.6	ESI+	Putative	46	1.0344	0.1320
528,1487@8,70	-	528.1487	8.70	-	M-H	4.2	ESI−	Putative	−35	1.2142	0.0275 *
PC(22:1)	C_30_H_58_NO_8_P	591.3900	18.76	4	M+Na	10.9	ESI+	Putative	−73	1.3013	0.0510 *
Kaempferiderhamnoside-(succinylglucoside)	C_32_H_36_O_18_	708.1902	7.97	2	M+H-H_2_O	5.9	ESI+	Putative	84	1.3239	0.0086
726,4535@18,99	-	726.4535	18.99	-	M-H	3.4	ESI−	Putative	−26	2.2514	0.0275 *
PS(39:7)	C_45_H_74_NO_10_P	819.5050	18.79	7	M+Na	5.3	ESI+	Putative	−96	1.2246	0.0151
PC(DiMe(9,3)/MonoMe(11,3))	C_45_H_79_NO_10_P	824.5442	17.98	6	M+H	2.6	ESI+	Putative	−115	1.0803	0.0510 *
PI(33:0)	C_42_H_81_O_13_P	825.5461	18.06	3	M+H	4	ESI+	Putative	−132	1.1234	0.0338 *

^a^ CV, coefficient of variation in the metabolites in the QC samples; ^b^ Change, percent change in the abundance of the specified comparison calculated as (case-control)/control) * 100, where the sign indicates the direction of change in the case group; ^c^ VIP, variable importance in projection; ^d^ *p* value * corresponding to the p values calculated by the Benjamini–Hochberg false discovery rate post hoc correction (FDR < 0.05). GM: global metabolomics, LC: liquid chromatography, QTOF-MS: quadrupole time-of-flight mass spectrometry.

**Table 2:** Means and standard deviations of the studied physicochemical properties and oxygen features for accepted samples (*n* = 6) and rejected samples (*n* = 6).

	Acceptance Group	
Parameter	Accepted Samples	Rejected Samples
Physicochemical		
Titratable acidity	59.93 ± 4.69 ^a^	72.19 ± 3.25 ^b^
Concentration [°Bx]	35.96 ± 0.56 ^a^	35.65 ± 0.26 ^a^
pH	4.79 ± 0.16 ^a^	4.76 ± 0.10 ^a^
Chroma *	27.92 ± 1.99 ^b^	25.09 ± 1.94 ^a^
a*	20.47 ± 1.30 ^b^	16.80 ± 1.39 ^a^
b*	18.96 ± 1.86 ^a^	18.63 ± 1.42 ^a^
L*	12.59 ± 1.14 ^a^	13.05 ± 0.78 ^a^
Hue	0.75 ± 0.04 ^a^	0.84 ± 0.02 ^b^
Oxygen and carbon dioxide		
O_2_—HS	7.63 ± 7.79 ^b^	0.30 ± 0.02 ^a^
CO_2_—HS	29.37 ± 16.89 ^a^	40.48 ± 5.35 ^b^
O_2_—Dissolved	0.24 ± 0.09 ^a^	0.27 ± 0.06 ^a^
O_2_—Saturation	3.08 ± 0.97 ^a^	3.76 ± 0.8 ^b^

* Chroma corresponds to the relationship between the color changes in the red (+a*) to green (−a*) ratio and the blue (−b*) to yellow (+b*) ratio, represented by a* and b*, respectively.

## Data Availability

The data presented in this study are available on request from the corresponding author (pending privacy and ethical considerations).

## References

[1] International Coffee Organization Coffee Market Report—June 2021. https://www.ico.org/Market-Report-20-21-e.asp.

[2] GlobalData (2019). Global Iced/RTD Coffee Drinks 2019.

[3] Torga G.N., Spers E.E., Florêncio de Almeida L., Spers E.E. (2019). Chapter 2—Perspectives of global coffee demand. Coffee Consumption and Industry Strategies in Brazil.

[4] Rocchetti G., Braceschi G.P., Odello L., Bertuzzi T., Trevisan M., Lucini L. (2020). Identification of markers of sensory quality in ground coffee: An untargeted metabolomics approach. Metabolomics.

[5] Ferreira V., Lopez R., Kerler J., Baggenstoss J., Moser M., Rytz A., Thomas E., Glabasnia A., Poisson L., Blank I. (2014). Advanced Analytical Sensory Correlation—Towards a Better Molecular Understanding of Coffee Flavor. Flavour Science.

[6] Villalón-López N., Serrano-Contreras J.I., Téllez-Medina D.I., Gerardo Zepeda L. (2018). An 1H NMR-based metabolomic approach to compare the chemical profiling of retail samples of ground roasted and instant coffees. Food Res. Int..

[7] Zapata J., Londoño V., Naranjo M., Osorio J., Lopez C., Quintero M. (2018). Characterization of aroma compounds present in an industrial recovery concentrate of coffee flavour. CyTA-J. Food.

[8] Sunarharum W.B., Williams D.J., Smyth H.E. (2014). Complexity of coffee flavor: A compositional and sensory perspective. Food Res. Int..

[9] Arana V.A., Medina J., Alarcon R., Moreno E., Heintz L., Schäfer H., Wist J. (2015). Coffee’s country of origin determined by NMR: The Colombian case. Food Chem..

[10] Consonni R., Polla D., Cagliani L.R. (2018). Organic and conventional coffee differentiation by NMR spectroscopy. Food Control.

[11] Hoyos Ossa D.E., Gil-Solsona R., Peñuela G.A., Sancho J.V., Hernández F.J. (2018). Assessment of protected designation of origin for Colombian coffees based on HRMS-based metabolomics. Food Chem..

[12] Gamboa-Becerra R., Hernández-Hernández M.C., González-Ríos Ó., Suárez-Quiroz M.L., Gálvez-Ponce E., Ordaz-Ortiz J.J., Winkler R. (2019). Metabolomic markers for the early selection of coffea canephora plants with desirable cup quality traits. Metabolites.

[13] Privat I., Foucrier S., Prins A., Epalle T., Eychenne M., Kandalaft L., Caillet V., Lin C., Tanksley S., Foyer C. (2008). Differential regulation of grain sucrose accumulation and metabolism in Coffea arabica (Arabica) and Coffea canephora (Robusta) revealed through gene expression and enzyme activity analysis. New Phytol..

[14] Gauglitz J.M., Aceves C.M., Aksenov A.A., Aleti G., Almaliti J., Bouslimani A., Brown E.A., Campeau A., Caraballo-Rodríguez A.M., Chaar R. (2020). Untargeted mass spectrometry-based metabolomics approach unveils molecular changes in raw and processed foods and beverages. Food Chem..

[15] Cavanna D., Righetti L., Elliott C., Suman M. (2018). The scientific challenges in moving from targeted to non-targeted mass spectrometric methods for food fraud analysis: A proposed validation workflow to bring about a harmonized approach. Trends Food Sci. Technol..

[16] Lamichhane S., Sen P., Dickens A.M., Hyötyläinen T., Orešič M. (2018). An Overview of metabolomics data analysis: Current tools and future perspectives. Compr. Anal. Chem..

[17] Sébédio J.L., Malpuech-Brugère C. (2016). Implementation of Foodomics in the Food Industry. Innovation Strategies in the Food Industry: Tools for Implementation.

[18] Diez-Simon C., Mumm R., Hall R.D. (2019). Mass spectrometry-based metabolomics of volatiles as a new tool for understanding aroma and flavour chemistry in processed food products. Metabolomics.

[19] Kim S., Kim J., Yun E.J., Kim K.H. (2016). Food metabolomics: From farm to human. Curr. Opin. Biotechnol..

[20] Rothwell J.A., Loftfield E., Wedekind R., Freedman N., Kambanis C., Scalbert A., Sinha R. (2019). A metabolomic study of the variability of the chemical composition of commonly consumed coffee brews. Metabolites.

[21] Quintero M., Velásquez S., Zapata J., López C., Cisneros-Zevallos L. (2021). Assessment of concentrated liquid coffee acceptance during storage: Sensory and physicochemical perspective. Molecules.

[22] Gigl M., Frank O., Barz J., Gabler A., Hegmanns C., Hofmann T. (2021). Identification and quantitation of reaction products from quinic acid, quinic acid lactone, and chlorogenic acid with strecker aldehydes in roasted coffee. J. Agric. Food Chem..

[23] Schoenauer S., Schieberle P. (2018). Structure–odor correlations in homologous series of mercapto furans and mercapto thiophenes synthesized by changing the structural motifs of the key coffee odorant furan-2-ylmethanethiol. J. Agric. Food Chem..

[24] Charles-Bernard M., Kraehenbuehl K., Rytz A., Roberts D.D. (2005). Interactions between volatile and nonvolatile coffee components. 1. Screening of nonvolatile components. J. Agric. Food Chem..

[25] Charles-Bernard M., Roberts D.D., Kraehenbuehl K. (2005). Interactions between volatile and nonvolatile coffee components. 2. Mechanistic study focused on volatile thiols. J. Agric. Food Chem..

[26] Frank O., Blumberg S., Kunert C., Zehentbauer G., Hofmann T. (2007). Structure determination and sensory analysis of bitter-tasting 4-vinylcatechol oligomers and their identification in roasted coffee by means of LC-MS/MS. J. Agric. Food Chem..

[27] Guerra S., Lagazio C., Manzocco L., Barnabà M., Cappuccio R. (2008). Risks and pitfalls of sensory data analysis for shelf life prediction: Data simulation applied to the case of coffee. LWT-Food Sci. Technol..

[28] Sopelana P., Pérez-Martínez M., López-Galilea I., de Peña M.P., Cid C. (2013). Effect of ultra-high temperature (UHT) treatment on coffee brew stability. Food Res. Int..

[29] Nicoli M.C., Calligaris S., Manzocco L. (2009). Shelf-life testing of coffee and related products: Uncertainties, pitfalls, and perspectives. Food Eng. Rev..

[30] Pérez-Martínez M., Sopelana P., De Peña M.P., Cid C. (2008). Application of multivariate analysis to the effects of additives on chemical and sensory quality of stored coffee brew. J. Agric. Food Chem..

[31] Sittipod S., Schwartz E., Paravisini L., Peterson D.G. (2019). Identification of flavor modulating compounds that positively impact coffee quality. Food Chem..

[32] Upadhyay R., Mohan Rao L.J. (2013). An outlook on chlorogenic acids-occurrence, chemistry, technology, and biological activities. Crit. Rev. Food Sci. Nutr..

[33] Sittipod S., Schwartz E., Paravisini L., Tello E., Peterson D.G. (2020). Identification of compounds that negatively impact coffee flavor quality using untargeted Liquid Chromatography/Mass Spectrometry Analysis. J. Agric. Food Chem..

[34] Pérez-Martínez M., Sopelana P., De Peña M.P., Cid C. (2008). Effects of refrigeration and oxygen on the coffee brew composition. Eur. Food Res. Technol..

[35] Smuda M., Glomb M.A. (2013). Fragmentation pathways during Maillard-induced carbohydrate degradation. J. Agric. Food Chem..

